# Aging With Multiple Sclerosis: Age-Related Factors and Socioeconomic Risks

**DOI:** 10.3389/fneur.2022.818652

**Published:** 2022-03-11

**Authors:** Malthe Faurschou Wandall-Holm, Mads Albrecht Andersen, Mathias Due Buron, Melinda Magyari

**Affiliations:** ^1^Danish Multiple Sclerosis Registry, Department of Neurology, Copenhagen University Hospital—Rigshospitalet, Glostrup, Denmark; ^2^Danish Multiple Sclerosis Center, Department of Neurology, Copenhagen University Hospital—Rigshospitalet, Glostrup, Denmark

**Keywords:** multiple sclerosis, aging, age-related risks, age-related factors, socioeconomic, socioeconomic outcomes

## Abstract

**Background:**

Studies have demonstrated an increasing mean age of the population with multiple sclerosis (MS). The association between increased age and socioeconomic outcomes has been investigated sparsely.

**Objective:**

The purpose of this study is to describe the demographic and socioeconomic status of the current Danish population of patients with MS according to age and to assess the age-related risks of no income or losing all income from earnings or receiving disability pension.

**Methods:**

The nationwide population-based Danish Multiple Sclerosis Registry provided data linked with the Danish Income Statistics Register and the Danish Rational Economic Agents Model (DREAM) database. The prevalence of socioeconomic milestones of the current MS population was compared with healthy controls and the risks of reaching socioeconomic milestones were assessed using cause-specific Cox models and cumulative incidence functions compared to healthy controls.

**Results:**

The current Danish population of patients with MS of working age (18–65 years of age) consists of 11,287 patients, of which 29.3% was older than 55 years. In 2018, 38.0% of all patients and 18.9% of controls had no income from earnings, whereas 30.5% of all patients and 7.7% of controls received disability pension. The risk of losing all income from earnings was higher for patients with MS with a hazard ratio (HR) peaking at of 4.0 (95% CI, 3.8–4.2) for the ages of 45–54 years. The risk of receiving disability pension was much higher for patients with MS peaking at a HR of 22.6 (95% CI, 20.9–24.4) for the ages of 25–34 years. Likewise, the absolute risks of both outcomes were higher for the patients with MS at all ages.

**Conclusion:**

Danish patients with MS are at a higher risk of losing all income from earnings and at a much higher risk of receiving disability pension compared with healthy controls.

## Introduction

Multiple sclerosis (MS) is considered a disease of the young adult, although numerous studies have reported an increasing mean age of the population with MS, and an increasing incidence of the elderly (1, 2).

The proportion of patients on disability pension and without income has been shown to increase drastically after disease onset (3, 4), and the work ability after a diagnosis of MS has been shown to be reduced (5). The socioeconomic burden of MS on society is substantial and appears more than 8 years before diagnosis with productivity decreases and social benefit payments making up the majority of this burden (6, 7). On an individual level, these consequences have negative implications for both mean income (3), but also the sense of personal contribution to society.

Aging in general is linked to challenges concerning physical and mental abilities. For patients with MS, these challenges are seen much earlier in life than for the rest of the population (8). A recent Danish study has reported an increased incidence of MS most pronounced at later ages of onset (1), and previous studies have found that older age at onset is associated with a later assignment of irreversible disability levels (9). However, the interplay between age and socioeconomic outcomes has not been extensively investigated.

The aim of this study was to investigate the association between age and socioeconomic decline in patients with MS in Denmark. We chose two reliable outcomes reflecting the functional capacity of the patient (10), no income or loss of income and disability pension, and compared prevalence, hazard ratios (HRs), and cumulative incidence across age groups with the background population of Denmark.

## Materials and Methods

### Study Design and Data Sources

We conducted a Danish nationwide observational study that consists of two parts: a cross-sectional study and a longitudinal study. Clinical data were obtained from the population-based nationwide Danish Multiple Sclerosis Registry (DMSR) (11) with information on patients with MS dating back to 1948. Demographic and socioeconomic data were obtained from national population-based registers: the Population Statistics Register (12), the Income Statistics Register (13) (ISR), and the Danish Rational Economic Agents Model (DREAM) (14). The primary outcomes were obtained from ISR and DREAM. The ISR contains data on income of all Danish citizens on an annual basis. DREAM contains data on all social transfer payments on a weekly basis. The unique personal identification code provided to all Danish citizens (12) enabled individual cross linkage between registers.

### Cross-Sectional Study

#### Study Population

The reference point in time of the cross-sectional study was January 1, 2019. All patients diagnosed with MS at January 1, 2019 were eligible for inclusion. The diagnosis of MS was made according to the Poser criteria before 2005 and the McDonalds criteria and subsequent revisions after 2005. To be included in the study, patients had to be alive, living in Denmark and be between 18 and 65 years of age (considered working age) at the reference point.

Disease duration was calculated as the time in years between the onset of MS (first clinical symptom) and January 1, 2019. The Expanded Disability Status Scale (EDSS) score was defined as the latest EDSS score within 2 years of the reference point. The MS phenotype was categorized as either relapsing-remitting MS (RRMS), secondary progressive MS (SPMS), or primary progressive MS (PPMS), and unspecified, assessed by a neurologist. Current treatment was categorized as receiving no treatment or with a disease modifying therapy (DMT) of moderate efficacy (azathioprine, dimethyl fumarate, glatiramer acetate, interferon-β, methylprednisolone cycles, peginterferon β-1a, and teriflunomide) or high efficacy (alemtuzumab, cladribine, daclizumab, fingolimod, hematopoietic stem cell transplantation, methotrexate, mitoxantrone, natalizumab, ocrelizumab, ofatumumab, rituximab, and treosulfan) at the reference point. Classification of DMTs as either moderate or high-efficacy was based on the ability to reduce relapse rates, reduction in MRI disease activity and disability accumulation (15).

Treatment coverage was calculated as years spent as either untreated, treated with a DMT of moderate efficacy or treated with a DMT of high efficacy divided by the disease duration.

Patients were grouped by age at the reference point into five categories: 18–24, 25–34, 35–44, 45–54, and 55–64. The intervals were chosen to reflect gradual changes in work ability during life.

#### Outcomes

No income was defined as having no income from personal earnings (including short-term sickness benefits) in the year of 2018 in the ISR. Disability pension was defined as having one or more transfer payments labeled “disability pension” in the year of 2018 in the DREAM register.

#### Statistical Analysis

Clinical characteristics were displayed as frequencies with corresponding percentages, mean values, and standard deviations (SD) or median values with interquartile ranges (IQR) as appropriate. The amount of missing data is displayed for each variable. To perform comparative analysis, we matched every included patient on sex and exact age as floored integers in a one-to-five manner. Controls were drawn from a random comparator sample containing 25% of the entire Danish population (excluding patients with MS). Prevalence ratios with confidence intervals (CIs) were calculated using a generalized linear model with a binomial distribution and a logarithmic link function. The calculation of a prevalence ratio is similar to that of a relative risk, but relative risk is a misnomer in the cross-sectional setting. There were no known missing data for outcomes.

### Longitudinal Study

#### Study Population

Patients with MS and all subjects in the 25% random sample of the Danish population were eligible for enrollment into the study population from January 1, 1992 to January 1, 2019. The period was chosen due to outcome availability in DREAM and ISR. Inclusion criteria were an age between 18 and 65 during the study period (to be at risk for the study outcome), being alive at January 1, 1992 (or born later), being an inhabitant in Denmark in January 1, 1992 (or born with Danish citizenship later), and not having received disability pension before enrollment (once granted, disability pension is considered permanent in Denmark). Patients diagnosed with MS during follow-up contributed risk time in both groups, changing status on the day of diagnosis. Diagnostic criteria for MS were according to the Poser criteria until 2005 and subsequently the McDonald criteria and their following revisions.

#### Outcomes

Loss of income was defined as the 2nd year without income for 2 consecutive years after having had at least 1 year with an income, identified in the ISR. This composite outcome was chosen, because we wanted loss of income to represent a weakening association with the labor market, and not a fluctuation due to temporary life situations: sick leave, leave of absence, long-term travels, etc. Disability pension was defined as the first occurrence of a transfer payment labeled “disability pension” in the DREAM register.

#### Statistical Analysis

The HRs and corresponding 95% CIs were assessed using cause-specific Cox regression models. The models used age as the underlying timescale (16). Subjects were entered into the model in a left truncated manner, only contributing risk time while observable. Using age as the timescale automatically ensured adjustment of the models for age. To account for the time dependency of exposure, subjects contributed risk time in the five age categories: 18–24, 25–34, 35–44, 45–54, and 55–64, changing categories as they aged during follow-up. Follow-up ended at the first occurrence of either a censoring event (death, emigration, turning 65 years old, being diagnosed with MS for the reference group, or end of follow-up) or an event (loss of income or disability pension, respectively). Exposure status (patient or control) was handled in a time-varying manner. Patients who were diagnosed during follow-up contributed risk time as controls until diagnosis and as patients after.

For the assessment of the absolute risks of losing all income from earnings or receiving disability pension, we fitted the same data using a non-parametric estimator of the cumulative incidence function taking competing risks into account (17). Death, emigration, and turning 65 years old were registered as competing risks, while developing MS (for the reference group) or not having reached an event by the end of follow-up was registered as censored. Exposure status changed as described in the previous paragraph.

The amount of missing data on outcomes was negligible (<1%), and analysis was performed on a complete case basis.

### Ethics, Approval, and Data Access

Observational register-based studies do not require informed consent or approval from the ethical committee in Denmark. The study was approved by the Danish Data Protection Agency. Danish data regulations dictate that access to data can only be obtained upon qualified request and approval by the Danish Data Protection Agency and the Danish Multiple Sclerosis Group.

Individual level data were pseudonymized. Table cells containing values representing data from <5 subjects (and neighbors allowing crosscell calculations) were censored due to Danish GDPR regulations. Data management and statistical analysis were performed on secure servers hosted by Statistics Denmark (18). All analyses were performed using SAS version 9.4 (SAS Institute Inc., Cary, NC, USA).

## Results

### Cross-Sectional Study

**Figure 1:**
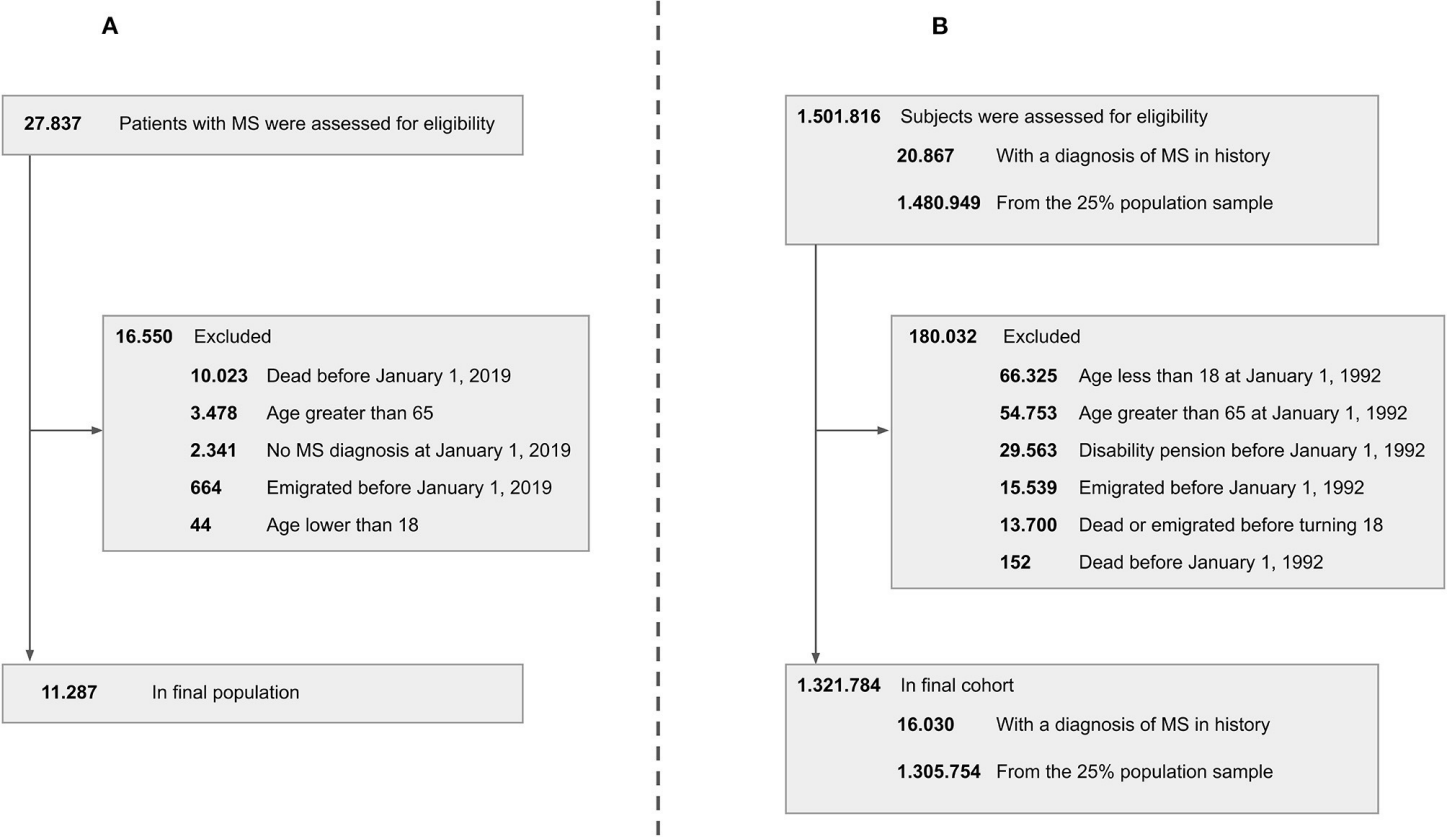
Patient disposition chart. Left side **(A)** cross-sectional study. Right side **(B)** longitudinal study. MS, multiple sclerosis.

A total of 27,837 patients in the DMSR were assessed for eligibility. Following exclusion of ineligible patients, 11,287 remained in the final population (Figure 1A). All variables were assessed in relation to the reference time point, January 1, 2019, as described in the Method section. The male-to-female ratio was ~1:2 in all age groups. The mean age at onset of the entire population was 32.8 years (SD, 9.6 years), and the median disease duration was 13.0 years (IQR, 13.5). The median EDSS score of the entire population was 2.0 (IQR, 2.0). We found an increase in median EDSS scores of 0.5 per age group (apart from the last, that increased by 1), starting at 1.0 (IQR, 2.0) among the 18–24-year age group and ending at 3.5 (IQR, 3.50) among the 55–64-year age group. The RRMS phenotype was predominant in the two youngest age groups, making up 89.6–89.9% of cases, while only being 54.9% of the cases in the oldest age group. In total, RRMS accounted for 74.8% of all patients in the population, whereas 7.7% had SPMS, 6.7% had PPMS, and 10.8% had an unspecified phenotype. The patients with an unspecified phenotype were older with a mean age of 52.7 years (SD, 9.6 years) and the majority, 85%, did not receive treatment.

At the reference point in time, 61.1% of the total population received treatment with a DMT, with 57.6% of these on DMT of moderate efficacy and 42.4% on DMT of high efficacy. Among patients with RRMS, a total of 73.8% received a DMT, with 57.5% of these on a DMT of moderate efficacy and 42.5% on a DMT of high efficacy.

For the RRMS population, the mean proportion of the disease duration without treatment was 0.45 (SD, 0.31), whereas 0.40 (SD, 0.31) was covered by drugs of moderate efficacy and 0.14 (SD, 0.24) by drugs of high efficacy. Results for the individual age groups can be found in Table 1.

**Table 1:** Clinical characteristics of the current population with MS in Denmark.

**Age**	**18–24**	**25–24**	**35–44**	**45–54**	**55–64**
	***n*** **= 197**	***n*** **= 1,310**	***n*** **= 2,695**	***n*** **= 3,779**	***n*** **= 3,306**
Female, *n* (%)	131 (66.5)	901 (68.8)	1,876 (69.6)	2,637 (69.8)	2,235 (67.6)
Age at onset, mean (SD)	18.10 (3.15)	23.70 (4.4)	29.3 (6.4)	34.4 (8.2)	38.5 (10.2)
Disease duration, median (IQR)	3.5 (3.5)	6.0 (6.0)	10.50 (10.0)	14.50 (13.0)	19.5 (16.0)
EDSS score, median (IQR)	1.0 (2.0)	1.5 (1.5)	2.0 (2.0)	2.5 (2.0)	3.5 (3.5)
**Phenotype**, ***n*** **(%)**					
RR	177 (89.8)	1,174 (89.6)	2,334 (86.6)	2,936 (77.7)	1,816 (54.9)
PP	9 (4.6)	65 (5.0)	132 (4.9)	218 (5.8)	335 (10.1)
SP	CENS	CENS	71 (2.6)	296 (7.8)	498 (15.1)
Unspecified	CENS	CENS	158 (5.9)	329 (8.7)	657 (19.9)
**Treatments grouped by efficacy, all patients**, ***n*** **(%)**					
No DMT	20 (10.2)	299 (22.8)	696 (25.8)	1,390 (36.8)	1,985 (60.4)
Moderate efficacy	81 (41.1)	507 (38.7)	1,021 (37.9)	1,432 (37.9)	935 (28.3)
High efficacy	96 (48.7)	504 (38.5)	978 (36.3)	957 (25.3)	386 (11.7)
**Has received treatment (If “No DMT” above)**, ***n*** **(%)**	16 (80.0)	216 (72.2)	449 (64.5)	793 (57.1)	797 (40.2)
**Treatment efficacy, patients with RRMS**, ***n*** **(%)**					
No DMT	15 (8.5)	237 (20.2)	507 (21.7)	761 (25.9)	689 (37.9)
Moderate efficacy	73 (41.2)	456 (38.8)	931 (39.9)	1,307 (44.5)	813 (44.8)
High efficacy	89 (50.3)	481 (41.0)	896 (38.4)	868 (29.6)	314 (17.3)
**Treatment coverage, patients with RRMS, mean (SD)**					
Untreated	0.37 (0.27)	0.41 (0.29)	0.42 (0.30)	0.45 (0.31)	0.52 (0.33)
Moderate efficacy	0.36 (0.32)	0.38 (0.30)	0.40 (0.31)	0.42 (0.31)	0.40 (0.32)
High efficacy	0.27 (0.32)	0.21 (0.28)	0.18 (0.25)	0.13 (0.25)	0.08 (0.18)

*CENS, Censored due to small cell values; EDSS, Expanded Disability Status Scale; RR, relapsing remitting; PP, primary progressive; SP, secondary progressive; DMT, disease modifying therapy; SD, standard deviation*.

For the comparative analysis, cases were matched with controls in a 1:5 ratio on sex and exact age, which yielded 56,435 controls. The percentage of patients having no income from earnings ranged from 30.0% in the 18–24-year age group to 54.3% in the 55–64-year age group, whereas for controls, it was 17.8 and 24.3%, respectively. In total, 38.0% of patients and 18.9% of controls had no income from earnings in 2018. The prevalence ratio peaked between the 55–64-year age groups at 2.2 (95% CI, 2.1–2.3), whereas the smallest prevalence ratio was found in the 25–34-year age groups at 1.5 (95% CI, 1.4–1.7).

The percentage of patients on disability pension was found to be increased from 2.0% in the youngest age group to 53.1% in the oldest, whereas the controls were found to be increased correspondingly from 1.0 to 13.8%. In total, 30.5% of patients and 7.7% of controls were on disability pension. The prevalence ratio was highest between the 35–44-year age groups at 5.4 (95% CI, 4.8–6.1) and lowest between the 18–24-year age groups at 2.0 (95% CI, 0.6–6.3), note the overlap of one in the confidence interval.

The results can be found in Table 2.

**Table 2:** Prevalence and prevalence ratios for socioeconomic outcomes according to age groups from cross-sectional analysis.

**Age, years**	**Events/patients (*n*/*n*, %)**	**Events/controls (*n*/*n*, %)**	**Prevalence ratio (95% confidence interval)**
**No income from earnings in 2018**			
18–24	59/197 (30.0%)	175/985 (17.8%)	1.7 (1.3–2.2)
25–34	347/1,310 (26.5%)	1,138/6,550 (17.4%)	1.5 (1.4–1.7)
35–44	713/2,695 (26.5%)	2,073/13,475 (15.4%)	1.7 (1.6–1.9)
45–54	1,376/3,779 (36.4%)	3,285/18,895 (17.4%)	2.1 (2.0–2.2)
55–64	1,796/3,306 (54.3%)	4,015/16,530 (24.3%)	2.2 (2.1–2.3)
**Receiving disability pension in 2018**			
18–24	4/197 (2.0%)	10/985 (1.0%)	2.0 (0.6–6.3)
25–34	98/1,310 (7.5%)	113/6,550 (1.7%)	4.3 (3.3–5.6)
35–44	523/2,695 (19.4%)	484/13,475 (3.6%)	5.4 (4.8–6.1)
45–54	1,292/3,779 (34.2%)	1,456/18,895 (7.7%	4.4 (4.2–4.7)
55–64	1,754/3,306 (53.1%)	2,285/16,530 (13.8%)	3.8 (3.7–4.0)

### Longitudinal Study

A total of 1,501,816 subjects were assessed for eligibility. Following exclusion of ineligible subjects, 1,321,784 remained in the final population (Figure 1B), of which 16,030 were patients with MS (at some point in the study period) and 1,305,754 were controls from the 25% random sample of the Danish population.

For the loss of income from earnings-analysis, total follow-up time was 107,243 person years for the patient group and 19,828,947 person years for the control group with a mean follow-up of 8.1 and 15.0 years, respectively. We observed 5,958 events in the patient group and 352,994 events in the control group. The highest HR of 4.0 (95% CI, 3.8–4.2) was found in the 45–54-year age group, whereas the lowest at 2.2 (95% CI, 2.1–2.3) was found in the 55–64-year age group.

For the disability pension-analysis, total follow-up time was 95,533 person years for the patient group and 21,300,413 person years for the control group with a mean follow-up of 6.8 and 16.1 years, respectively. We observed 5,347 events in the patient group and 98,200 events in the control group. The highest HR of 22.6 (95% CI, 20.9–24.4) was found in the 25–34-year age group, whereas the lowest at 5.5 (95% CI, 5.2–5.9) was found in the 55–64-year age group. Results from both Cox regression analyses are presented in Table 3.

**Table 3:** HRs for socioeconomic outcomes according to age groups from longitudinal analysis.

**Age, years**	**Hazard ratio**
	**(95% confidence interval)**
**Loss of all income for 2 consecutive years**	
18–24	3.6 (3.0–4.3)
25–34	3.0 (2.8–3.2)
35–44	3.1 (3.0–3.3)
45–54	4.0 (3.8–4.2)
55–64	2.2 (2.1–2.3)
**Receiving disability pension**	
18–24	19.5 (15.3–24.8)
25–34	22.6 (20.9–24.4)
35–44	12.1 (11.4–12.7)
45–54	7.9 (7.5–8.3)
55–64	5.5 (5.2–5.9)

**Figure 2:**
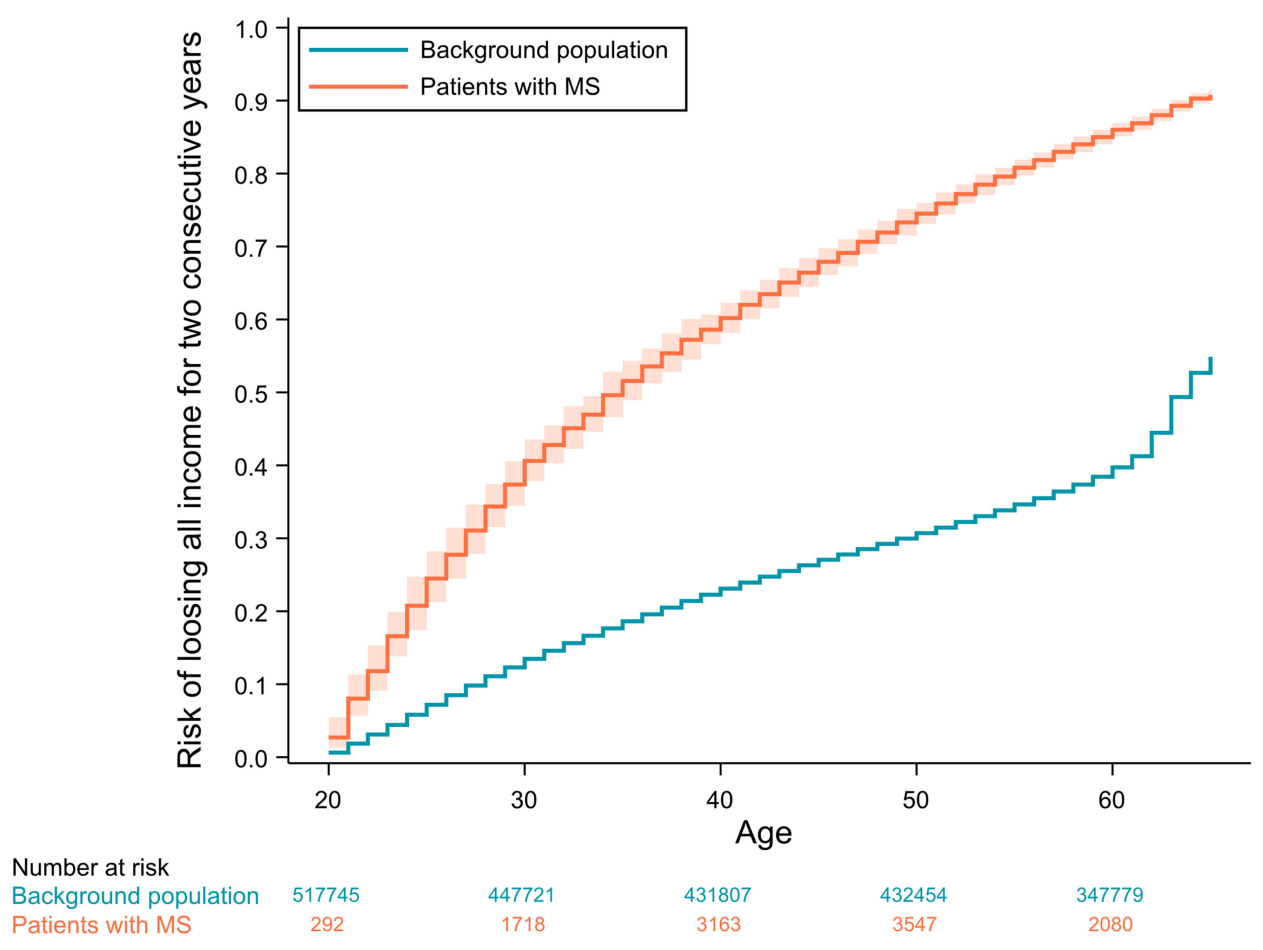
Cumulative incidence curves of losing all income for 2 consecutive years with 95% confidence intervals adjusted for competing risks (age ≥ 65, emigration or death). Confidence intervals for the background population are not visible due to values very close to the incidence estimates.

**Figure 3:**
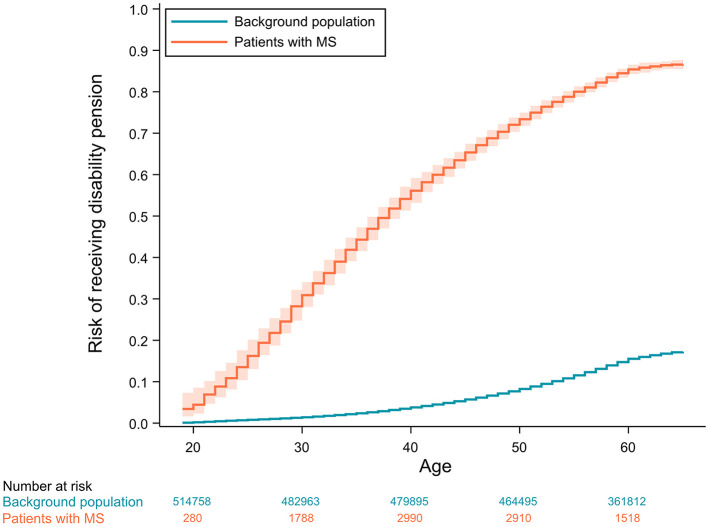
Cumulative incidence curves of receiving disability pension with 95% confidence intervals adjusted for competing risks (age ≥ 65, emigration or death). Confidence intervals for the background population are not visible due to values very close to the incidence estimates.

The absolute risks of losing all income from earnings or receiving disability pension as a function of age are displayed in Figures 2, 3, respectively.

## Discussion

In this study, we describe a nationwide population of patients living with MS in Denmark and their socioeconomic status measured by loss of income from earnings and disability pension according to age categories.

We found that the Danish population alive and of working age consists of 11,287 patients with MS, of which approximately two-thirds are female, similar to what is reported in other populations (19). The mean age at onset was around 33 years with a median disease duration of 13 years—not surprisingly, both are increasing with age. We found a median EDSS score of 2 for the whole population, ranging from 1 for the youngest age group to 3.5 for the oldest. This was expected since the EDSS score is known to be highly age-dependent due to disability accumulating over time (9).

Further, elderly patients tend to have increased activity of neurodegenerative pathways and less effective neuro-repair processes compared with younger patients—processes that are known to be significant determinants of disability accumulation in MS (20, 21).

The distribution of MS phenotypes was found to be almost exclusively RRMS for the youngest patients, whereas patients in the oldest age group of 55–64 years had a higher prevalence of progressive phenotypes: PPMS (10.1%), RRMS (55.0%), and SPMS (15.0%). Obviously, SPMS is more frequently seen at higher ages as it takes time for the disease to progress to the secondary progressive phase (21), but the incidence of PPMS has also been shown to increase at higher ages of disease onset (22). This is especially prevalent in male patients, who more often present with PPMS and a higher age at onset (22).

Regarding the use of DMT for patients with RRMS, we saw that high efficacy treatment was most widely used in the younger patients, and the proportion of moderate efficacy treatment increased with increasing age. At the same time, there was an increase of not being on treatment with increasing age. In the four youngest age categories, the increase was modest, while changing category from 45–55 to 55–64 saw a relative increase of 46.2% of patients being untreated. The mean proportion of the disease duration covered by treatment increased with decreasing age from 48% among the 55–64 years old compared to 63% among the 18–24 years old. The most likely explanation for this difference is earlier diagnosis and subsequently earlier treatment starts, increased availability of treatments and a more aggressive therapeutic approach to disease management.

The data illustrate that the aging patient group represents a clinical challenge with regard to treatment. A quarter of the population in the age group of 55–64 years display a distinct progressive phenotype that, despite recent advances in therapeutical options (23, 24), are still mainly left untreated. Among the patients of the highest age group still clinically regarded as having a relapsing remitting phenotype, we see a treatment drop-off accelerating with increasing age. The reason for the decrease in DMT use has not been extensively investigated and is most likely a combination of overlapping explanations, such as contraindications, patient preference, progression, or stable disease without relapses which has poor evidence on treatment effectiveness in the aging MS population (25).

In the clinical setting, no distinct event or biomarker indicates a progression from the relapsing remitting to the secondary progressive phenotype, rather it is a gradual process that at some point reaches a diagnostic threshold. The transition implies an underlying shift in pathological pathways from peripherally induced acute inflammation to chronic inflammation and neurodegenerative processes (26, 27). Since the current arsenal of treatment options for MS is targeting acute inflammation, the disease becomes increasingly treatment refractory. The combination of perceived reduced disease activity combined with less efficacy of available treatments might make the clinician and patients less prone to choose treatment. Another driver could be a higher risk of adverse events in the elderly population (25, 28). This theory can also explain the relative preference of moderate effective DMTs at higher ages, which implies that emphasis is less upon treatment efficacy but rather tolerability in elderly patients. It is also worth to note that many of the patients in the highest age group were diagnosed around the time of the advent of disease modifying therapies. When the earliest drugs, interferon-βs, became available in Denmark in the late nineties, national treatment guidelines limited the immunomodulatory treatment to patients with two or more relapses per year. As such, only patients with high disease activity had access to treatment in those years.

When assessing the prevalence of no income from earnings in the cross-sectional analysis, we found a significantly increased prevalence for patients with MS across all age categories. This finding is like that of previous studies from Sweden showing a higher percentage with at least one record of no income from salaries within 10 years after diagnosis, and in general, a lower income after diagnosis compared with healthy controls (4, 29). The differences between patients and controls were considerably smaller than those seen for receiving disability pension, which indicate that income loss for 1 year is a more frequent occurrence in the background population, in turn increasing the validity of our application of a composite income–outcome in the longitudinal analysis. There is a surprisingly large number of events in both the MS and the control group among those aged 18–24 having no income, which is mainly due to students not having an income from earnings. The prevalence ratio should remain robust since education is free and with equal access in Denmark.

Looking at the cumulative incidence functions of the absolute risk of losing all income from earnings for 2 consecutive years, the risk is diverging until the control population reach their fifties and the risk starts converging, due to healthy controls also beginning to lose their income from earnings. Interestingly, the same age-related acceleration of income-loss is not seen for the patients with MS. This is possibly due to a selection of socioeconomically robust patients in the higher age groups that have managed to maintain an income up until this point. These patients, who have not lost their income this late in life, are likely systematically different from the rest of the patient population, in that they might be more resilient or have an income from earnings not as dependent on physical or mental ability.

When assessing the risk of disability pension, we found that the current Danish population of patients with MS of working age had a 22.8% higher prevalence (30.5 vs. 7.7%) of receiving disability pension compared with controls. These differences were statistically significant across all age categories except the youngest, probably due to low overall occurrence of the outcome in this category. The Social Pension Law of Denmark allows for granting of disability pensions at ages below 25, but only under extreme circumstances.

In the longitudinal analysis, we found a substantial difference in the HRs for receiving disability pension for all age groups. A peak in HR of disability pension was found at ages of 25–34 years. The HRs of income loss did not display the same rise and fall and remained stationary during midlife. The cumulative incidence curve clearly supports a massively increased absolute risk of receiving disability pension in patients with MS, with patients showing a similar risk of receiving disability pension at the age of 25 as the controls do at the age of 65. The ever-increasing divergence of the risk is in line with other studies, which shows that the risk of losing employment is related to higher age but also increased disability, lower education, higher age at onset, longer disease duration, and more fatigue (30, 31).

Our MS population was nationwide and population-based. The analysis would be strengthened by the application of multivariate adjustment, specifically with the addition of EDSS scores; however, the untreated patients are not seen frequently in the clinic, and thus, many do not have recent EDSS scores available. Another limitation is the absence of descriptive socioeconomic variables such as level of education and type of labor on the current population with MS in Denmark. An addition of these would have made comparisons with countries of dissimilar structures of social legislation and health care easier. Citizens of Denmark are provided free education and free access to health care making the Danish population very homogenous. Thus, the comparisons within the Danish population in this study are only prone to small amounts of related confounding.

A strength of this study is the large amount of data we have in the DMSR linked to other population-based registries, which makes the results less prone to random variation, giving us the possibility to calculate estimates with high precision. Another strength is that independently collected data in large Danish registries and databases can be merged by the Danish unique personal identification code. Our data on income and disability pension have a virtually complete capture rate and it is representative of the whole Danish population due to the nationwide nature (14, 18).

Our results support the hypothesis that MS drastically worsens the socioeconomic status of patients. EDSS has been used as an outcome measure for decades, and it is easy to compare between patients cross-sectionally. However, the impact of disease experienced by the patients may raise other concerns such as fatigue, sleep, or their ability to maintain their job; important aspects of life are not captured by EDSS.

Socioeconomic status is not only dependent on income from earnings and disability pension. We hypothesize that our results may represent a tendency of MS influencing many socioeconomic factors of the patients, so that these trends could be shown for other aspects such as care and assistance at home or education after diagnosis, though more research is needed to reveal the exact nature of associations between MS and other socioeconomic factors. Using socioeconomic parameters as outcomes in MS research is warranted as these are highly affected by the disease while having substantial consequences for patients with MS on a personal level. Further, receiving disability pension or losing all yearly income constitute relevant, somewhat “hard” endpoints, that are likely very reflective of the functional capacity of patients. Such studies are well-suited and feasible to perform using Danish nationwide MS data allowing for linkage to registries holding socioeconomic data.

We found that the Danish patients with MS are at a higher risk of losing all income from earnings and at a much higher risk of receiving disability pension compared with healthy controls. Both risks were shown to increase drastically by age. Although our results focus on the ability of patients to maintain a job, we hypothesize that MS also influences many other socioeconomic factors in life.

## Data Availability Statement

The original contributions presented in the study are included in the article/supplementary material, further inquiries can be directed to the corresponding author.

## Ethics Statement

Ethical review and approval was not required for the study on human participants in accordance with the local legislation and institutional requirements. Written informed consent for participation was not required for this study in accordance with the national legislation and the institutional requirements.

## Author Contributions

MW-H, MA, and MB: design, conceptualization, methodology, software, data analysis, visualization, drafting, and revision for intellectual content. MM: design, conceptualization, methodology, drafting, revision for intellectual content, supervision, funding acquisition, and resources. All authors contributed to the article and approved the submitted version.

## Funding

This study was financially supported by the Danish Multiple Sclerosis Society, a non-governmental, patient organization.

## Conflict of Interest

MM has served on Scientific Advisory Board for Biogen, Sanofi, Roche, Novartis, Merck, and AbbVie, has received honoraria for lecturing from Biogen, Merck, Novartis, Sanofi, and Genzyme, and has received research support and support for congress participation from Biogen, Genzyme, Teva, Roche, Merck, and Novartis. The remaining authors declare that the research was conducted in the absence of any commercial or financial relationships that could be construed as a potential conflict of interest.

## Publisher's Note

All claims expressed in this article are solely those of the authors and do not necessarily represent those of their affiliated organizations, or those of the publisher, the editors and the reviewers. Any product that may be evaluated in this article, or claim that may be made by its manufacturer, is not guaranteed or endorsed by the publisher.
